# Improving RNAi in the Brown Marmorated Stink Bug: Identification of target genes and reference genes for RT-qPCR

**DOI:** 10.1038/s41598-018-22035-z

**Published:** 2018-02-27

**Authors:** Kanakachari Mogilicherla, Jeffrey L. Howell, Subba Reddy Palli

**Affiliations:** Department of Entomology, University of Kentucky, Lexington, Kentucky 40546 USA

## Abstract

The brown marmorated stink bug (BMSB) is native to Asia and recently invaded the USA. RNA interference (RNAi) is a gene silencing mechanism in which the introduction of double-stranded RNA (dsRNA) inhibits gene function by degrading target mRNA. In dsRNA stability assays, the dsRNases present in the hemolymph and salivary gland secretions of BMSB showed lower activity than those in the hemolymph of *Heliothis virescens*. We evaluated six housekeeping genes (*18S rRNA*, *EF1-α*, *Actin*, *Ubiquitin*, *60S RP* and *β-Tubulin*) across dsRNA treatments (injection and feeding) in nymphs and adults of BMSB and identified *18S rRNA* and *60S RP* as the best genes to use as a reference in reverse-transcriptase quantitative PCR (RT-qPCR). Homologs of 13 genes that were shown to function as effective RNAi targets in other insects were identified and evaluated by injecting dsRNA targeting these homologs into BMSB adults. Five out of 13 dsRNAs tested caused more than 70% mortality by seven days after injection of dsRNA. Feeding dsRNA targeting five of these genes (*IAP, ATPase, SNF7, GPCR*, and *PPI*) to nymphs caused more than 70% mortality by three of the five dsRNAs tested. These data suggest that feeding dsRNA causes target gene knockdown and mortality in BMSB.

## Introduction

The brown marmorated stink bug (BMSB), *Halyomorpha halys*, is a highly polyphagous insect that feeds on more than 120 plants including field crops, trees, vegetables, and ornamentals^1^. Native to eastern Asia, the BMSB was first discovered in Pennsylvania, then rapidly spread in the Mid-Atlantic region of the USA; damaging crops and distressing homeowners due to their overwintering behavior^2^. In a recent special issue of Journal of Pest Science, three review articles^3–5^ summarized chemical ecology and chemical and biological control methods of this pest. In addition, 23 original papers have reported on the latest research on biology and management of BMSB^6^.

After the discovery of RNA interference (RNAi) in the nematode, *Caenorhabditis elegans* and demonstration of its functioning in insects, several groups started using this technology in basic research as well as for the development of methods to control insect pests and disease vectors^7–10^. Double-stranded RNA (dsRNA) is delivered to insects in a variety of ways, including injection, feeding or through transgenic plants or microorganisms such as bacteria^7,10–12^. RNAi efficiency varies among insects depending on the species, delivery method and genes targeted. For example, RNAi works efficiently and is systemic in most of the coleopteran insects tested^7,10,13^. However, RNAi efficiency is quite variable in most other insects, including those belonging to Lepidoptera, Hemiptera, and Diptera^14,15^. Differences in degradation of dsRNA by dsRNases, transport into and within cells, processing of dsRNA and differences in expression and structures of proteins involved in RNAi are among the major contributors to differential RNAi efficiency among insects^15,16^. Comparison of dsRNA transport and processing between lepidopteran and coleopteran cells and tissues demonstrated that dsRNAs are taken up by coleopteran cells and are processed into siRNAs, resulting in silencing of target genes. Conversely, lepidopteran cells take up dsRNAs, but they are accumulated in acidic bodies and hence not processed into siRNAs. Thus, the target genes are not silenced efficiently^15^. Recent studies by Yoon *et al*.^17^ identified the acidic bodies where dsRNAs are accumulated as early and late endosomes in the lepidopteran insect, *Spodoptera frugiperda*; suggesting that endosomal entrapment is one of the major contributors to RNAi inefficiency.

The efficiency of RNAi is also variable among hemipteran insects tested so far. RNAi appears to work well in heteropteran insects but does not perform as effectively in homopteran insects such as aphids and whiteflies. However, some published reports showed successful silencing of target genes by delivering dsRNA to homopteran insects by injection, feeding or through transgenic plants^18–20^. In BMSB, Bansal *et al*.^21^ injected adult BMSB with dsRNA targeting the catalase gene and observed knockdown. In addition, knockdown of genes coding for Juvenile hormone acid O-methyltransferase (JHAMT) and vitellogenin (Vg) in BMSB nymphs after orally delivering dsRNA targeting these genes through green beans was also reported recently^22^. However, in these studies, a significant mortality of BMSB after knockdown of these genes was not observed.

To identify target genes that could be used in the development of RNAi-based control methods for BMSB, we identified homologs of 13 genes that worked well as RNAi targets in other insects and screened them by injecting dsRNA targeting these genes in adult BMSB. Five out of 13 dsRNAs tested caused significant corrected percent mortality. Feeding dsRNA targeting three of these genes (*IAP, SNF7* and *PPI*) to BMSB nymphs caused more than 70% mortality. The data included in this study demonstrate that feeding dsRNA causes knockdown of target genes and mortality in BMSB.

## Materials and Methods

### Rearing of BMSB and Tobacco budworm

BMSB were collected from South and North Farms of the University of Kentucky between July and September 2017. Insects were reared in a greenhouse following the published methods (see Medal *et al*.)^23^. BugDorm-2120 insect rearing cages (61 × 61 × 61 cm) were kept in a greenhouse and maintained at a photoperiod of 16:8 h L:D, 26 °C ± 2, and 50–55% RH. Ornamental plants (*Peperomia obtusifolia Variegata*) were provided for shelter and resting, green bean and sweet corn plants were provided for oviposition, and organic green beans and peanuts were offered to the insects as dietary supplements. The tobacco budworm (TBW) *Heliothis virescens* was reared on an artificial diet as described previously^24^.

### Isolation of total RNA and cDNA synthesis

Total RNA was isolated from BMSB adults using TRI Reagent® RT (Molecular Research Center Inc., Cincinnati, OH). The DNase1 was used to remove the contaminating DNA from the total RNA (Ambion Inc., Austin, TX). The purified RNA was stored at −80 °C until further use. The integrity and quality of total RNA were analyzed on 1.2% agarose gels and quantified in Nanodrop 2000 Spectrophotometer (Thermo scientific). Two micrograms of total RNA for each sample was used for cDNA synthesis using M-MLV Reverse Transcriptase (Invitrogen).

### Gene amplification, purification and dsRNA synthesis

The cDNA was used as a template to amplify fragments of target genes using gene-specific primers (Table S1). PCR reaction contained 1 μl of cDNA template, 1 μl of 10 μM each primer, 25 μl of Taq 2× Master Mix (NEB, USA) in a total volume of 50 μl. PCR conditions used are as follows: 5 min at 95 °C for initial denaturation followed by 30 sec at 95 °C, 30 sec at 55 °C and 45 sec at 68 °C for 35 cycles and final extension 10 min at 68 °C. The amplified products were analyzed on 1% agarose gels and purified using PCR purification kit (Qiagen). The purified PCR products were quantified using a Nanodrop 2000 Spectrophotometer (Thermo Scientific) and stored at −20 °C until further use. Double-stranded RNA (dsRNA) targeting gene coding for green fluorescence protein (GFP, control) and 13 target genes were synthesized by using MEGAscript® T7 RNAi kit (Ambion, USA) following manufacturer’s instructions. The integrity of the dsRNA was analyzed on 1% agarose gels, and the concentration was determined by Nanodrop 2000 Spectrophotometer (Thermo scientific).

### Collection of hemolymph from BMSB and Tobacco budworm

Hemolymph was collected from BMSB and last instar larvae of Tobacco budworm. The larvae and adults were placed on ice, the forelegs of larvae and thoracic region of adults were pierced with a needle and hemolymph was collected into 1.5 ml tubes containing Phenylthiourea (Sigma-Aldrich). The hemocytes and other cell debris were removed from hemolymph by centrifugation and supernatant were used for dsRNA stability assay.

### Collection of watery saliva from BMSB

Watery saliva was collected from BMSB adults as described Peiffer and Felton^25^. BMSB adults were chilled on ice for five minutes then placed ventral side up and observed under a microscope. BMSB secreted saliva from the tip of the beak when they returned to room temperature. This saliva was collected into a 1.5 ml tube placed on ice by using 10 µl pipet tip containing 3 µl of 1× PBS buffer.

### ^32^P- labelling of dsGFP

The ^32^P-labelled dsGFP was prepared using MEGA script® T7 RNAi kit (Ambion, USA) as described recently^15^.

### dsRNA stability assay

Total protein in the hemolymph and watery saliva was estimated by Bradford method^26^. Different dilutions of hemolymph (1.2, 0.6, 0.3 and 0.15 mg/ml), and saliva (4.8, 2.4, and 1.2 mg/ml) were prepared, and 250 ng of dsRNA was added to the hemolymph and saliva. After an hour of incubation at room temperature (RT), samples were mixed with 2 μl of 6× loading dye, loaded and analyzed on 1% agarose gel and photographed by using AlphaImager Gel Imaging System (Alpha Innotech, San Leandro, CA). The hemolymph samples were also incubated 1hr with 8000 CPM ^32^P-UTP labelled dsGFP and run on 16% polyacrylamide-8M urea gel using 1X TBE buffer. Gels were washed and fixed with a solution containing 0.5X TBE buffer, 10% ethanol, and 10% methanol. The gels were dried using the gel drier. Gels were exposed to Phosphor-Imager screen and scanned using Typhoon FLA 9500 Laser Scanner (GE Healthcare Life Sciences).

### Injection of dsRNA into BMSB adults

One microgram of dsRNA in 5 μl of distilled water was injected into the thoracic region of adults using insulin syringe (1/2 cc U-100 insulin syringe). Each replicate used 10 adults, and 3–4 replicates were used for each treatment, and the experiments were repeated twice. Adults were collected at three days after injection for determining knockdown efficiency, and mortality was recorded at seven days post-injection.

### Feeding of dsRNA

Certified organic green beans (*Phaseolus vulgaris* L.) were used for feeding dsRNA as described previously^22^. Lean green beans were selected to ensure their fit in the 2 ml Eppendorf tubes. The beans were washed three times with ddH2O and trimmed from the calyx end to a total length of 5 cm. The beans were immersed in a capless 2 ml microcentrifuge tube containing 300 μl nuclease free water and 20 μg of dsRNA (added daily for 3 days). The tubes were sealed with parafilm to prevent evaporation of the solution and to prohibit insects from entering the solution. Beans were immersed in water containing dsRNA 3 hr prior to feeding. The tubes were then placed in a solid platform to keep them upright and enclosed within magenta jars (Sigma-Aldrich). Second instar BMSB nymphs were starved overnight prior to feeding. 15 nymphs were released per magenta jar containing one green bean in a microcentrifuge tube containing dsRNA (20 µg daily for three days) solution. Samples were collected for RT-qPCR study on the 4^th^ day after initiation of feeding and mortality was recorded on the 7^th^ day.

### Selection of candidate reference gene, RT-qPCR primers and analysis of amplification efficiency

The candidate reference genes were selected based on their previous reports in other insects. These include 1*8S ribosomal RNA* (XM_014421522.1), *Elongation factor 1 alpha* (XM_014438029.1), *Actin* (XM_014431329.1), *Ubiquitin* (XM_014429239.1), *60S ribosomal protein* (XM_014430141.1) and *Beta Tubulin* (XM_014438117.1). For each candidate reference gene, BLASTN and BLASTX were carried out in NCBI (http://www.ncbi.nlm.nih.gov/) and CDS region was identified using ExPASy translate tool (http://web.expasy.org/translate/). The IDT PrimerQuest software (http://eu.idtdna.comwebcite) was used for designing primers. The primer sets were checked for amplification specificity and annealing temperature. The specificity of the PCR amplified product was characterized by electrophoresis. Primer sets that amplified a single-specific product were chosen for RT-qPCR amplification efficiency test (Fig. S1).

### Reverse-transcriptase quantitative real-time PCR (RT-qPCR)

RT-qPCR experiments used StepOnePlus™ Real-Time PCR System (Applied Biosystems, USA). A 10 μl reaction volume [containing, 2 μl of diluted cDNA (1:2), 5 μl of iTaq™ universal SYBR® Green Supermix (Bio-Rad), 0.2 μl of each primer] was used. The RT-qPCR was performed under the following conditions: an initial denaturation step for 20 sec at 95 °C, followed by 40 cycles of amplification with 5 sec of denaturation at 95 °C, 30 sec of annealing and extension at 55 °C. The melt curve was obtained by heating the amplicon from 60 to 95 °C. A non-template control (NTC) was also included in each run for each gene. The *18S rRNA gene* was used as a housekeeping gene to normalize the RT-qPCR data.

### Statistical analysis

The stability levels of the six candidate reference genes from BMSB were determined using four statistical algorithms, geNorm^27^, NormFinder^28^, BestKeeper^29^ and RefFinder^30^. The corrected percent mortality was calculated based on Schneider-Orelli’s formula^31^. Double delta Ct (^ΔΔ^Ct) method was used for RT-qPCR data analysis^32^. A one-tailed t-test was used to compare the mean of a single variable.

## Results and Discussion

### Comparison of dsRNase activity

**Figure 1:**
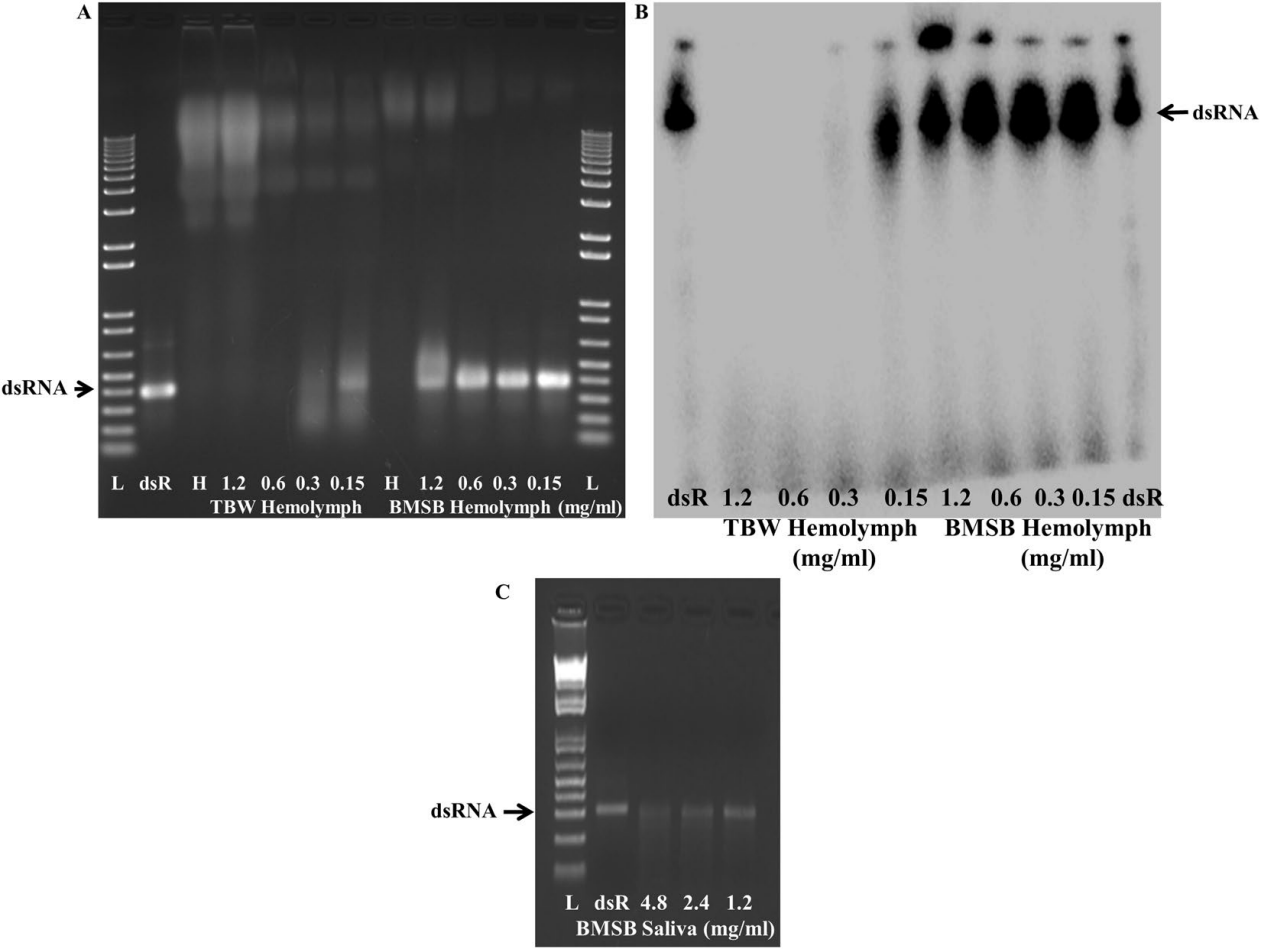
Analysis of dsRNA stability in Tobacco budworm and BMSB hemolymph. Agarose gel electrophoresis analysis of dsRNA degradation products exposed to Tobacco budworm (TBW), *Heliothis virescens* (Lepidoptera) and BMSB, *Halyomorpha halys* (Hemiptera) hemolymph and salivary gland secretions. (**A**) About 250 ng of dsGFP was exposed to various concentrations (1.2–0.15 mg/ml) of hemolymph from TBW or BMSB for an hour at room temperature, and the products were resolved on 1% agarose gels, and the gels were stained with ethidium bromide. L, 1 kb plus DNA ladder; dsR, 270 ng dsGFP alone and H, hemolymph of TBW or BMSB. (**B**) About 8000 CPM of ^32^P-labeled dsGFP was exposed to various concentrations of hemolymph (1.2–0.15 mg/ml) for an hour at room temperature, and the products were resolved on 16% polyacrylamide 8 M urea gels. The gels were dried and exposed to a PhosphorImager screen, and the image was scanned using a PhosphorImager. dsR, labeled dsGFP. (**C**). The BMSB saliva was collected as described in the Materials and Methods section. Protein concentration was determined and various concentrations (4.8–1.2 mg/ml) of saliva added to 250 ng dsGFP and incubated at room temperature for an hour. The products were resolved on 1% agarose gel, and the gel were stained with ethidium bromide. L, 1 kb plus DNA ladder and dsR, 270 ng dsGFP alone.

For the systemic spread of fed dsRNAs from the midgut to other tissues, the dsRNAs have to travel through hemolymph where they may encounter dsRNases. Our previous studies showed that the activity of dsRNases is higher in the hemolymph of *Heliothis virescence* (Lepidoptera) where RNAi does not work well, as opposed to their activity in the hemolymph of *Leptinotarsa decemlineata* (Coleoptera) where RNAi works well^15^. To determine if the dsRNase activity in the hemolymph of BMSB could be detrimental to RNAi in this insect, we compared dsRNase activity in the hemolymph of BMSB and TBW (Lepidoptera). About 250 ng of dsGFP was incubated with serial dilutions of hemolymph and watery saliva (diluted with 1xPBS in a total volume of 5 μl based on total protein concentration) for an hour at room temperature. After incubation, the dsRNA samples were analyzed by 1% agarose gel or 16% polyacrylamide 8 M urea gels. The hemolymph collected from TBW degraded dsRNA at a concentration (0.3 mg/ml) or higher (Fig. 1A). The more sensitive method using ^32^P-labeled dsRNA also showed similar results (Fig. 1B). However, the hemolymph and saliva collected from BMSB did not completely degrade dsRNA even at 1.2 mg/ml concentration (Fig. 1A–C). These data suggest that the dsRNase activity in the BMSB is lower than that of TBW; thus, RNAi efficiency may be higher in BMSB when compared to that in TBW. The previous report suggested that higher levels of dsRNases are one of the major factors contributing to inefficient RNAi in pea aphid^16^. Recently, Bansal *et al*.^21^ demonstrated silencing of catalase gene by injection of dsRNA in BMSB. In another study, Ghosh *et al*.^22^ developed a green bean-based feeding method and showed effective silencing of target genes coding for JHAMT and Vg by feeding dsRNA. ^32^P labelled dsRNA injected into BMSB adults is processed into siRNA^33^, suggesting that these insects possess machinery to take up dsRNA and process to siRNA. The results from the previous reports and our finding of lower levels of dsRNase activity in the hemolymph and saliva of BMSB suggest that the RNAi could work well in BMSB and may be a viable option for controlling this invasive pest.

### Selection of candidate reference genes

To determine the knockdown efficiency of target gene after dsRNA administration, the mRNA or protein levels of target genes need to be quantified. Since antibodies are not available for most of the target genes being tested, quantification of mRNA levels is the most commonly used method. To quantify mRNA levels by RT-qPCR, a reliable reference gene is a prerequisite. Although some reference genes have been identified in BMSB, in the preliminary studies, we did not find them stable across the dsRNA treatments in our experiments. Therefore, we conducted experiments to identify reference genes.

To identify suitable reference genes, six genes (*Ubiquitin*, *Elongation factor*, *60S RP*, *Actin*, *18S rRNA* and *β-Tubulin*) were selected based on previous reports in other insects. Information on the selected reference genes is shown in Table S2. Melt curve analysis was performed to confirm the specific amplification of each reference gene and a single peak with no visible primer-dimer formation and genomic DNA contamination was observed and no signals were detected in the nontemplate controls (NTC) (Fig. S1). The candidate reference genes, primer sequences, and amplicon sizes are shown in Table S3.

**Figure 2:**
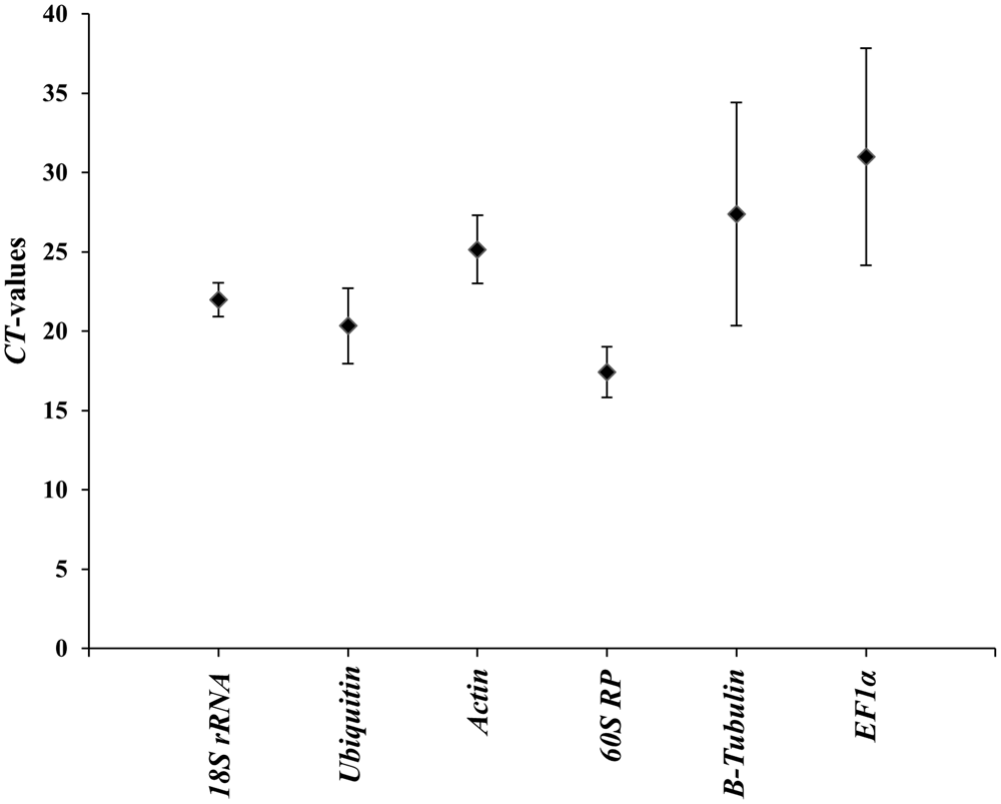
Identification of stable reference genes. Variability of the Ct values for six reference genes in all samples (Injection and feeding dsRNA) of BMSB nymphs and adults tested. Total RNA was isolated after injection/feeding dsRNA. The RNA was converted to cDNA, and the cDNA and gene-specific primers were used in RT-qPCR to determine Ct values. Mean ± SD of Ct values are shown.

Expression levels of six candidate reference genes were measured using RNA isolated from dsRNA injected or fed BMSB adults and nymphs. The Ct values for these genes varied from 15–40 (Fig. 2). *18S* rRNA, Ubiquitin, actin and *60S* RP showed lower variation in their Ct values when compared to those of the other two genes tested (Fig. 2). The geNorm algorithm^27^ was used to calculate the average expression stability value (*M*-value), using Ct values of each gene among the dsRNA treatments. The genes with the lowest *M*-value were considered as the most stable. The geNorm analysis identified *18S rRNA* and *60S RP* as the most stable genes (Table 1) across the dsRNA treatments. The stability of the six housekeeping genes was further analyzed using the NormFinder algorithm^28^. The NormFinder analysis of the datasets estimated the stability of genes based on intra- and intergroup variation. The genes with fewer stability values were considered to be the most stable. This program also identified *18S rRNA* and *60S RP* as the most stable genes across the dsRNA treatments (Table 1).

**Table 1:** Ranking of the candidate housekeeping genes according to their stability value by geNorm, NormFinder, and BestKeeper analysis. M, the gene expression stability measure; SD, standard deviation value; SV, stability value; GM, Geomean value and R, Ranking.

Gene Name	geNorm		NormFinder		BestKeeper		ΔCT		Comprehensive		
	M	R	SV	R	SD	R	SD	R	GM	R	
Adult dsRNA injected samples											
18s rRNA	0.733	1	0.33	1	0.52	1	3.91	1	1	1	
Ubiquitin	0.821	2	0.384	3	0.99	4	4.05	3	3.46	4	
60S RP	0.733	1	0.37	2	0.64	3	4.01	2	2.06	2	
Actin	0.948	3	0.33	1	0.61	2	4.12	4	2.83	3	
β-Tubulin	3.901	4	8.65	4	8.01	6	9.33	5	5.23	5	
EF1α	5.877	5	9.248	5	6.15	5	9.83	6	5.73	6	
Nymph dsRNA fed samples											
18s rRNA	0.356	1	0.18	1	0.51	2	1.51	1	1.19		1
Ubiquitin	0.356	1	0.47	2	0.43	1	1.57	2	1.41		2
60S RP	0.656	2	0.48	3	0.52	3	1.65	3	3		3
Actin	2.105	5	1.12	6	2.52	6	3.57	6	6		6
β-Tubulin	1.097	3	1.96	5	1.36	5	2.3	5	4.73		5
EF1α	1.372	4	3.44	4	1.14	4	2.03	4	4.23		4
Combined adult injected and nymph fed samples											
18s rRNA	0.893	1	0.446	1	0.89	1	3.75	1	1.19		1
Ubiquitin	1.152	2	1.425	2	2.07	4	4.06	3	3.22		3
60S RP	0.893	1	0.446	1	1.33	2	3.79	2	1.41		2
Actin	1.823	3	1.96	3	1.37	3	4.47	4	3.72		4
β-Tubulin	5.357	5	7.692	5	4.61	6	8.27	6	6		6
EF1α	3.903	4	7.129	4	5.92	5	7.81	5	5		5

The descriptive statistics of all six housekeeping genes used in the study were computed by the BestKeeper algorithm^29^. *18S rRNA* showed standard deviation (SD) values less than 1 an indicator of the consistent and stable performance. The coefficient of variation (*CV*) of housekeeping genes ranged from 0.89% for *18S rRNA* to 4.61% for *β-Tubulin* (Table 1).

RT-qPCR is a powerful technique to study the gene expression due to its high sensitivity, accuracy, specificity, and reproducibility. During RNA isolation, cDNA conversion and assembling reactions variability could be introduced. This could be countered using appropriate reference genes. Also, use of multiple reference genes is important because using a single reference gene may not be sufficient to control variability across all treatments^34^. In the present study, six reference genes were selected and validated in adult and nymph stages treated with different dsRNAs by injection and feeding and the data were analyzed by four statistical algorithms: geNorm, NormFinder, BestKeeper, and RefFinder. The results showed that *18S rRNA*, *60S RP* and *Ubiquitin* are the most stable reference genes in nymphs (Supp. Figs S3B and S4B; Table S5); *18S rRNA* and *60S RP* in adults, (Supp. Figs S3A and S4A; Table S4) and when both nymphs and adults were compared, the *18S rRNA* and *60S RP* are identified as the most stable genes (Table 1). In a recent study, ten housekeeping genes were evaluated for their stability across various treatments in BMSB and showed that *ARP8* and *Ubiquitin E4A* as the most stable genes across tissues and developmental stages treatments tested. *Ubiquitin* is the common gene identified in both these studies and may be the most stable genes to target across developmental stages and treatments. These studies also confirm the previous finding that the same housekeeping gene may not work well for all stages and treatments. Therefore one needs to identify one or more reference genes for the specific treatments compared by RT-qPCR.

### Screening of target genes and feeding RNAi

**Figure 3:**
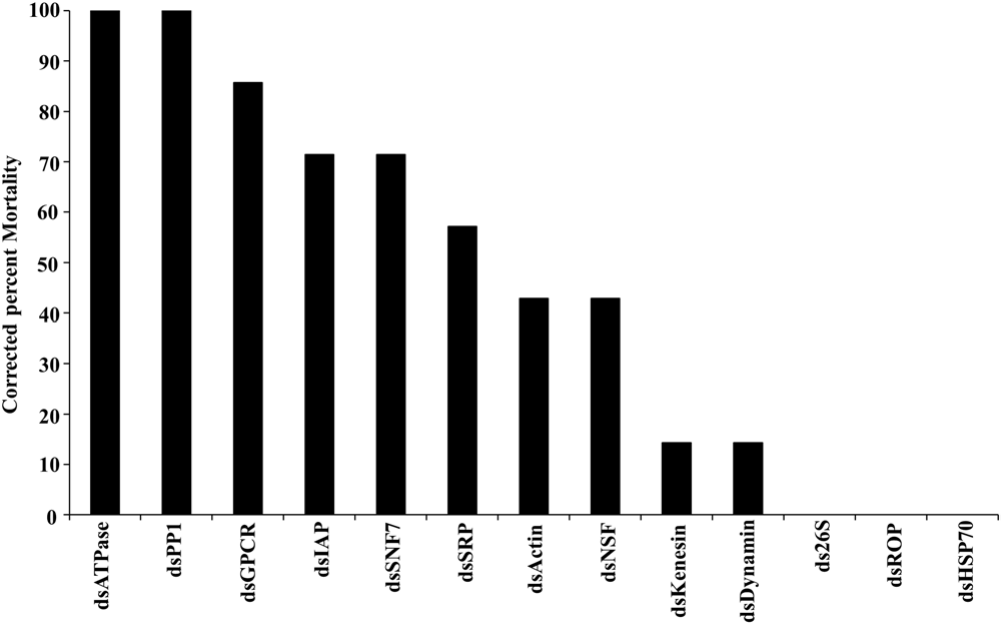
Screening of RNAi target genes in BMSB adults by injection of dsRNA. One microgram of dsRNA targeting each of the 13 select genes, ATPase (Putative ATPase N2B, Acc. no: XM_014420983.1); PP1 (Serine/threonine-protein phosphatase PP1-beta catalytic subunit, Acc. no: XM_014431150.1); GPCR (G protein-coupled receptor 161-like, Acc. no. XM_014438952.1); IAP (Baculoviral IAP repeat-containing protein 7-B-like, Acc. no: XM_014435389.1); SNF7 (Charged multivesicular body protein 4b, Acc. no: XM_014427464.1); SRP (Signal recognition particle 54 kDa protein, Acc. no: XM_014419857.1); Actin (Actin-5C, Acc. no: XM_014433214.1); NSF (Alpha-soluble NSF attachment protein, Acc. no: XM_014434606.1); Kanesin (Uncharacterized Kanesin, Acc. no: XM_014426093.1.); Dynamin (Dynamin, Acc. no: XM_014433358.1); *26*S (*26*S protease regulatory subunit 6B, Acc. no: XM_014421516.1); ROP (Protein ROP, Acc. no. XM_014418690.1); HSP70 (Heat shock 70 kDa protein cognate 3, Acc. no: XM_014425902.1) was injected into BMSB adults. The mortality was recorded on the 7th day after injection. dsRNA targeting gene coding for GFP was used as a control.

**Figure 4:**
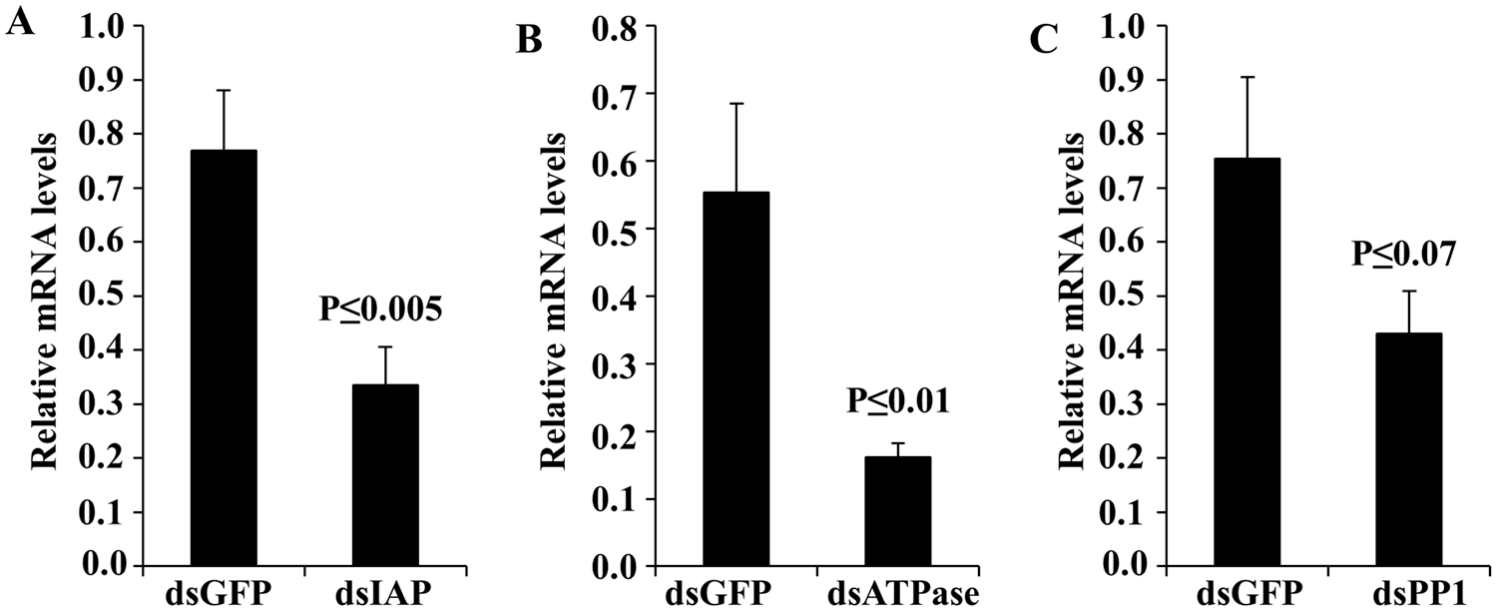
Knockdown efficiency determined by RT-qPCR in dsRNA injected BMSB. One microgram of dsRNA targeting IAP, ATPase, PPI or GFP (control) was injected in BMSB adults. Total RNA was isolated on the 3rd day after injection of dsRNA. The RNA was converted to cDNA, and the cDNA and gene-specific primers were used to quantify mRNA levels of IAP, ATPase, and PPI using RT-qPCR. The *18*S rRNA gene was used to normalize expression. The mean of relative mRNA levels and SE (n = 3–5) in dsGFP and dsIAP (**A**), dsATPase (**B**) or dsPP1 (**C**) injected insects are shown. A one-tailed t-test was used to compare the means of a single variable.

To identify target genes that could be used for RNAi-mediated control of BMSB, we screened 13 genes that are known to cause mortality in other insects after their knockdown by RNAi. 1.0 μg of dsRNA targeting each of the selected genes or gene coding for GFP as a control were injected into each BMSB adult. At seven days after injection, the mortality was recorded. Five out of the 13 dsRNAs tested caused more than 70% mortality by seven days after injection (Fig. 3). Quantification of mRNA levels of three of these genes (IAP, PP1 and ATPase) using RT-qPCR showed 40–75% knockdown in the expression of these target genes in insects injected with dsRNA targeting each of these genes (Fig. 4). Considerable differences in the efficacy of the 13 dsRNAs in causing mortality of BMSB was observed. This may be due to the differences in function of these target genes. Also, differences in knockdown efficiency of the target genes by the dsRNAs used could also account for some of the differences in the efficacy of dsRNAs tested.

**Figure 5:**
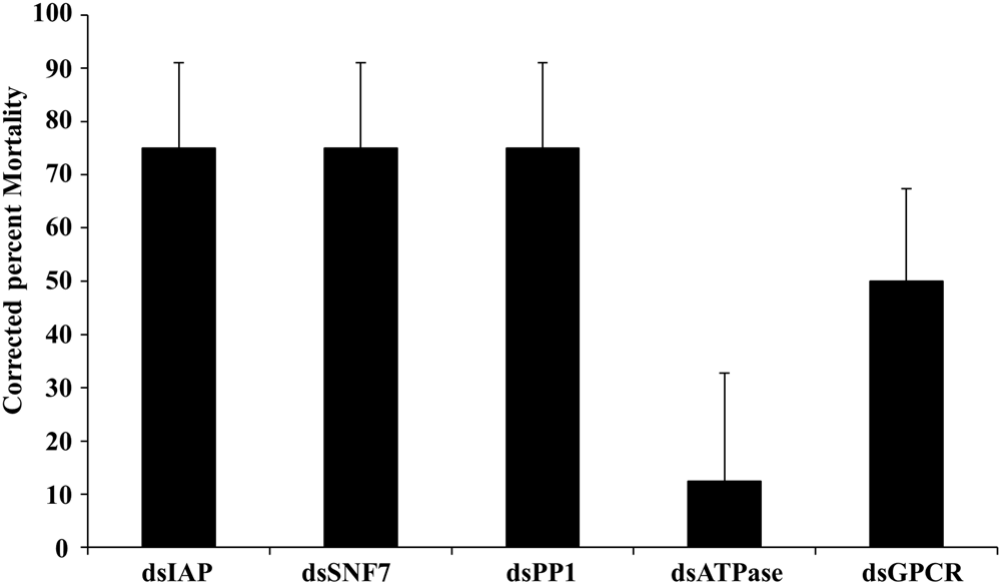
Feeding dsRNA causes mortality in BMSB. Certified organic beans were inserted in 2 ml tubes containing 300 µl of nuclease-free water and 20 µg of dsRNA (20 µg of dsRNA in water was added to the tube on the second and third day). Fifteen 2^nd^ instar nymphs were fed on each bean for seven days. The mortality was recorded on the 7^th^ day, and the corrected percent mortality was calculated using Schneider-Orelli’s formula. Mean + S.E (n = 3) are shown. The asterisk reflects significant differences in mortality rate (ANOVA, Student-Newman-Keuls Method, P < 0.05).

**Figure 6:**
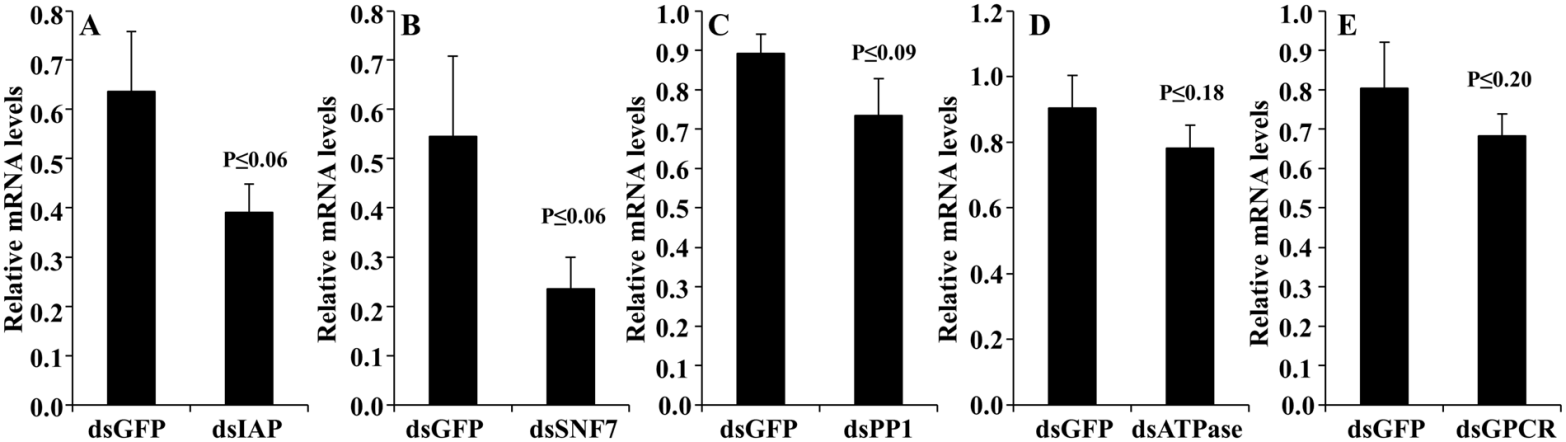
Knockdown efficiency determined by RT-qPCR in dsRNA fed BMSB. Twenty micrograms of dsRNA targeting IAP, SNF7, PPI, ATPase, GPCR or GFP (control) was fed to BMSB nymphs on each day for three days. On the fourth day, the nymphs were fed on beans. Total RNA was isolated on the 4th day after initiation of feeding dsRNA. The RNA was converted to cDNA, and the cDNA and gene-specific primers were used to quantify mRNA levels of IAP, SNF7, ATPase, PPI and GPCR using RT-qPCR. The *18S* rRNA mRNA levels were used to normalize expression. The mean of relative mRNA levels and SE (n = 3–4) in dsGFP and dsIAP (**A**), dsSNF7 (**B**), dsPPI (**C**), dsATPase (**D**) or dsGPCR (**E**) fed nymphs are shown. A one-tailed t-test was used to compare the mean of a single variable.

BMSB frequently feed on fruit crops and beans in agricultural systems using their needle-like stylets by alternate salivation and ingestion^25^. BMSB are highly attracted to green beans and are major pests of many bean varieties. Green beans (*Phaseolus vulgaris* L.,) were selected for delivery of dsRNA. Slender green beans were trimmed from the calyx, inverted and immersed into dsRNA solution (0.066 μg/μl) in a 2 ml microcentrifuge tube. Each bean was placed in a magenta vessel, and 15 nymphs were released per vessel (Fig. S2). Second instar nymphs were starved overnight prior to exposure to dsRNA. Five dsRNAs targeting *IAP, ATPase, SNF7, GPCR*, and *PPI* were tested in feeding RNAi bioassay. Three of the five dsRNAs tested caused more than 70% corrected mortality (Fig. 5). RT-qPCR analysis showed 60–20% knockdown in the expression of five of these genes in BMSB nymphs fed on dsRNA targeting each of these genes (Fig. 6). These data suggest that feeding dsRNA causes significant knockdown of target gene and mortality in BMSB.

In this present study, oral delivery of dsRNA through green beans caused mortality in BMSB, confirming the effectiveness of RNAi in BMSB and demonstrating that feeding dsRNA could induce RNAi and mortality in this insect. Both injection and feeding of dsIAP caused mortality, and gene knockdown data suggests that the *IAP* gene may be one of the best target genes to control the BMSB using RNAi. The *IAP* gene from *Bombyx mori* was identified and shown to function as a caspase inhibitor to block apoptosis^35^. The functioning of RNAi in the tarnished plant bug, *Lygus lineolaris* was demonstrated using the *IAP* gene as the target by delivering dsIAP to the nymphs and adults through microinjection^36^. The dsIAP treated insects showed a significant reduction in the lifespan when compared with those injected with control dsRNA. The I*AP1* gene was identified in *Aedes aegypti* and showed that the gene product inhibits both initiator and effector caspases^37^. In Aag-2 cell, five genes coding for *IAPs* (1, 2, 5, 6 and 9) were identified. Treating these cells with dsRNA targeting these genes caused a significant reduction in the mRNA levels of target genes but only dsIAP1 induced apoptosis phenotype^38^. In Lepd-SL1 cell line, a gene coding *IAP1* was identified and exposing these cells to dsIAP1 induced apoptosis^39^. These investigators used *IAP1* to develop an assay to identify genes critical of RNAi pathway in these cells. Exposing Ledp-SL1 cells to dsRNA targeting RNAi pathway genes followed by dsIAP1 showed that five genes (*Argonaute-1, Argonaute-2a, Argonaute-2b, Aubergine and V-ATPase 16* *kDa subunit 1* and *Vha16*) are essential for successful RNAi in these cells. Recently, Rodrigues *et al*.^40,41^ targeted *IAP1* gene in two invasive forest pests, *Agrilus planipennis* and *Anoplophora glabripennis* and showed significant knockdown of *IAP1* gene after injection of dsIAP1.

The previous two reports and the data included in this paper showed that RNAi works in BMSB and feeding dsRNA could be a viable control option for this pest. The next major challenge is the delivery of dsRNA: What is the best way to get dsRNA to BMSB in the field?, Expression in crop plants, delivery through green beans drenched with dsRNA and placed as bait stations in the field, dsRNA spray on the foliage and dsRNA applied to the soil are just a few of the possible routes of application that need to be evaluated.

## Electronic supplementary material

Supplementary Information
