# Supplementary material for: Improving RNAi in the Brown Marmorated Stink Bug: Identification of target genes and reference genes for RT-qPCR
Source: Sci Rep. 2018 Feb 27;8:3720. doi: 10.1038/s41598-018-22035-z (PMC5829169; doi:10.1038/s41598-018-22035-z)
Supplement: Supplementary file 1 — Supplementary Information [file 41598_2018_22035_MOESM1_ESM.pdf]

Improving RNAi in the Brown Marmorated Stink Bug: Identification of target genes and reference genes for RT-qPCR

Kanakachari Mogilicherla, Jeffrey L. Howell and Subba Reddy Palli*

Department of Entomology, University of Kentucky, Lexington, Kentucky 40546, USA

Email: (K.M.) kmo246@g.uky.edu; (J.H.) Jeff.Howell@uky.edu; *Correspondence addressed to (S.R.P.): rpalli@email.uky.edu

Supplementary Information:

Figure S1. Melting curve analysis of qPCR amplification of six housekeeping genes (*18S rRNA*, *Ubiquitin*, *60S RP*, *Actin*, *β-Tubulin* and *EF1α*) using gene-specific primers

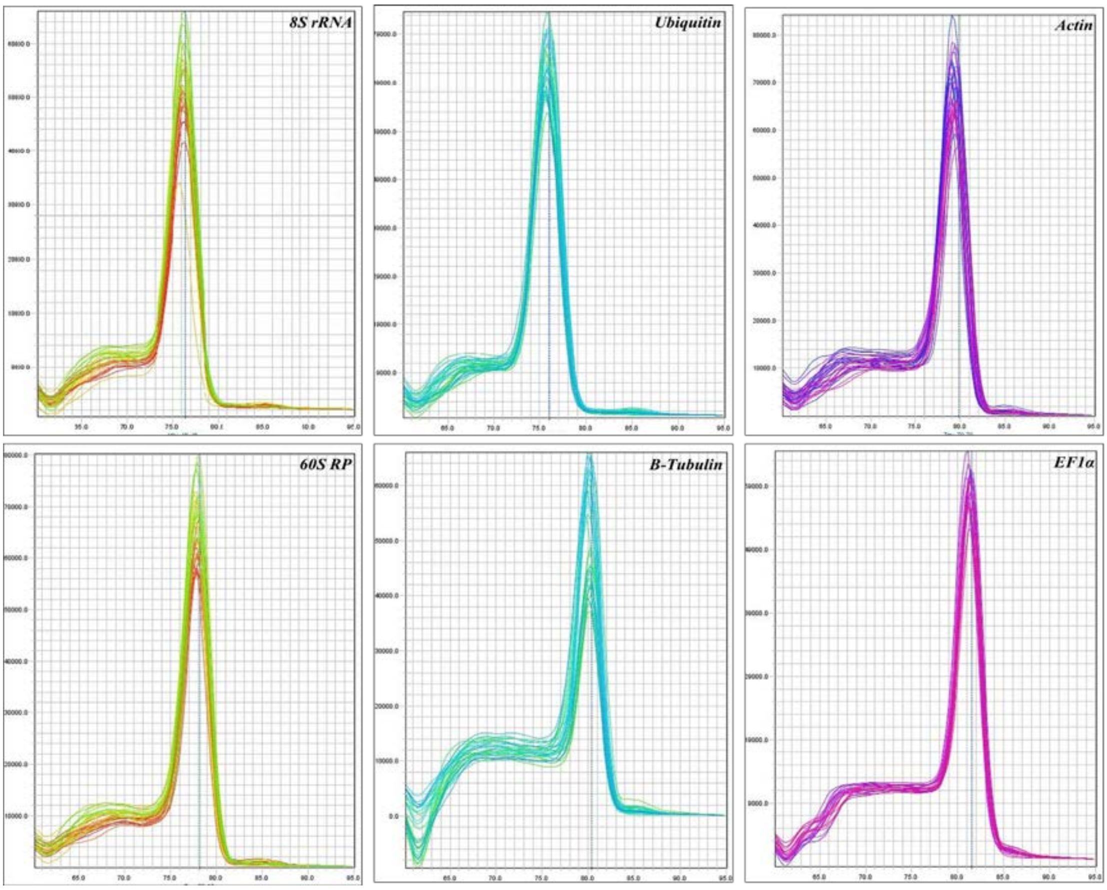

Figure S2. **Setup for Stink bug nymph's feeding assay**. Certified organic green beans were inserted in 2 ml Eppendorf tubes containing 300 µl of nuclease free water with 20 µg of dsRNA (20 µg each day for 3days), fixed to a solid support and placed in magenta jars. 20 µg of five different target genes (*IAP*, *SNF7*, *ATPase*, *PP1* and *GPCR*) dsRNA was fed to 2nd instar BMSB nymphs (15 nymphs per group), whereas, the dsGFP was used as a control.

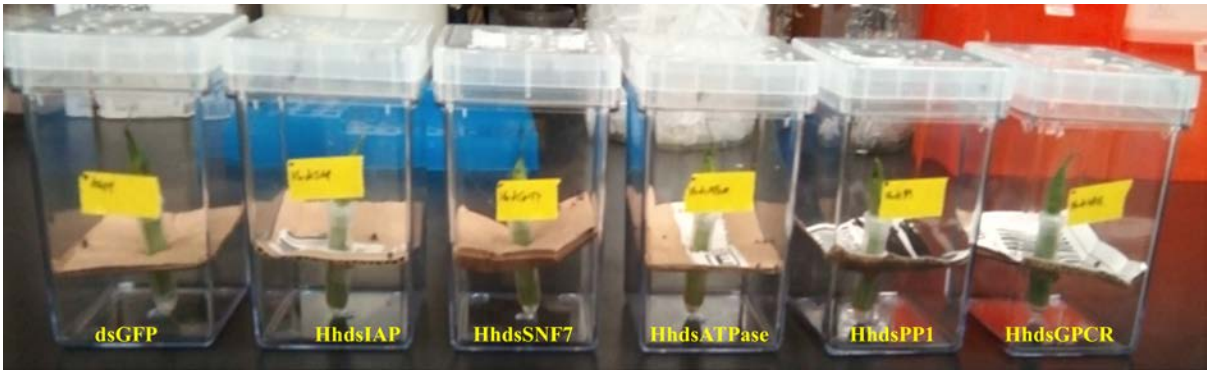

Figure S3: Expression stability analysis of each candidate reference gene in different dsRNA treated samples using the geNorm tool. Gene expression stability graph based on average expression stability values (M-value), using stepwise exclusion process. A lower M-value

indicates, higher gene stability. The direction of the arrow indicates the most and least stable housekeeping genes. (A) dsRNA injected adult samples; (B) dsRNA fed nymph samples.

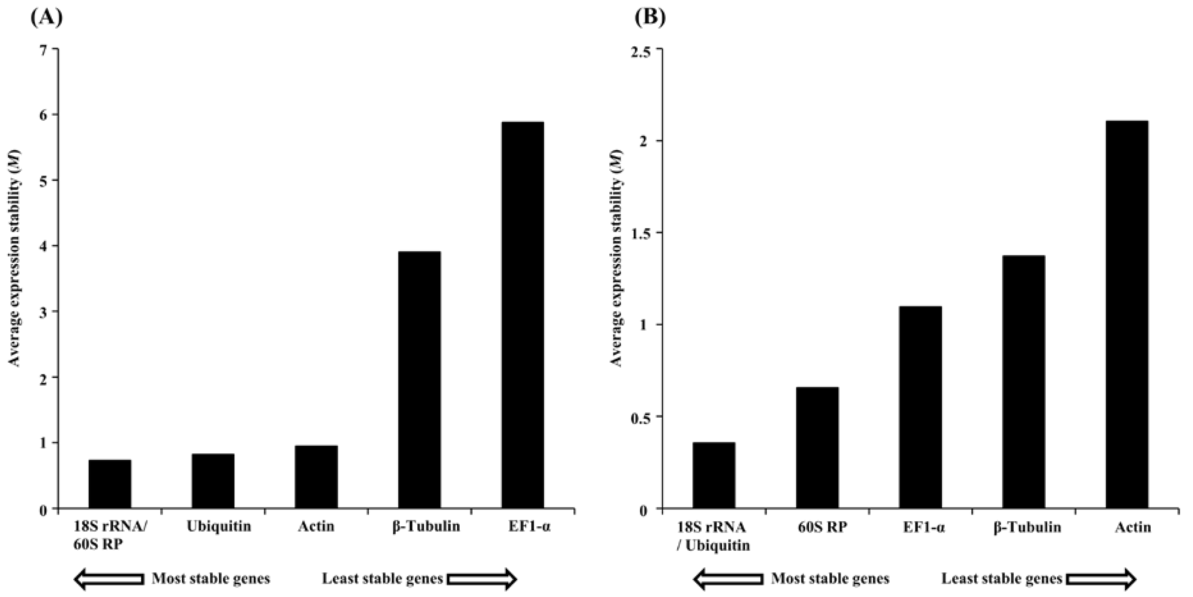

Figure S4: NormFinder analysis of the datasets estimated the stability value of all tested genes based on intra and inter-group variation. The genes with lower stability values were considered to be the most stable, whereas with highest stability values were ranked as the least stable genes. (A) dsRNA injected adult samples; (B) dsRNA fed nymph samples.

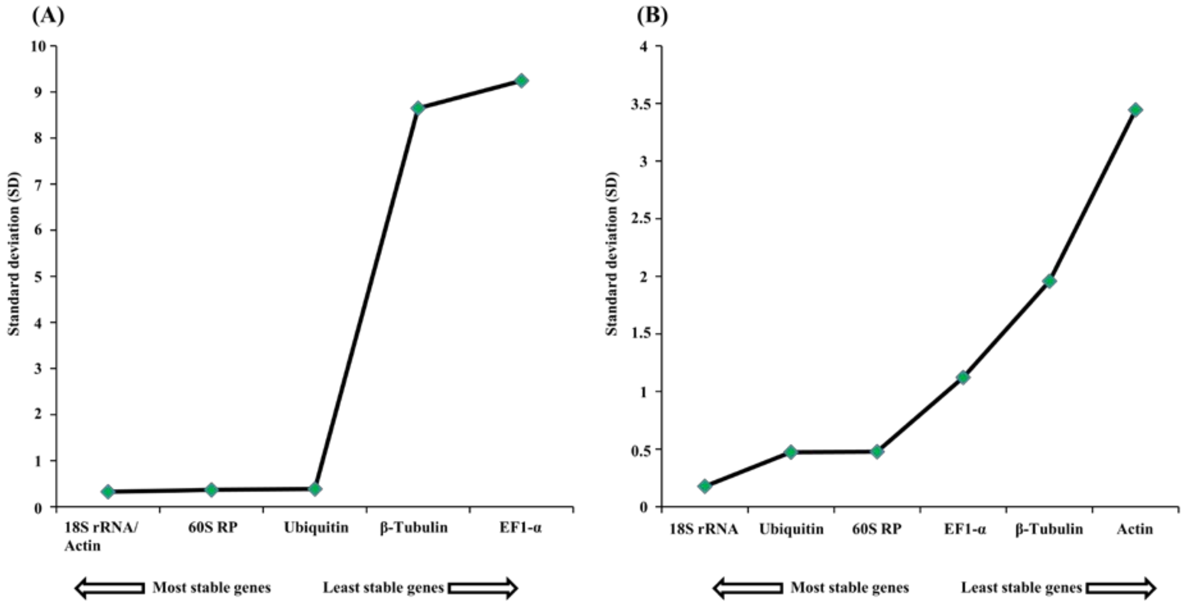

Table S1. List of primers used for dsRNA synthesis

Gene name	Accession No. (*Halyomorpha halys*)	Primer sequence (5'-3')	Tm (°C)	Product size (bp)
Vacuolar-sorting protein (SNF7)	XM_014427464.1	F: TAATACGACTCACTATAGGGTCTGAGGAACAAGAGGTTAG<br>R: TAATACGACTCACTATAGGGCTTCAGTTCGTCGTCCTCTTC	60<br>60	471
IAP repeat-containing protein 7-B-like (IAP)	XM_014435389.1	F: TAATACGACTCACTATAGGGTATGCCCATTGGCCAGAAA<br>R: TAATACGACTCACTATAGGGCTAGAAGCTCCGGACTAACAATAC	60<br>60	343
Actin-5C (Actin)	XM_014433214.1	F: TAA TAC GAC TCA CTA TAG GGTTAAGGGACCGACAAAGA<br>R: TAA TAC GAC TCA CTA TAG GGCCCAACTCACCGCTAGAATAA	63<br>64	453
Signal recognition particle 54 kDa protein(SRP)	XM_014419857.1	TAATACGACTCACTATAGGGATGCGTTGCGTTCGTTAAG<br>TAATACGACTCACTATAGGGTCCACACCATCTTGAGCAATAA	63<br>64	497
Alpha-soluble NSF attachment protein (NSF)	XM_014434606.1	TAATACGACTCACTATAGGGACACTGACATGGGAAGGTTTAC<br>TAATACGACTCACTATAGGGCCACTGATCCAGTCGTGAAATAG	64<br>64	494
Protein ROP (ROP)	XM_014418690.1	TAATACGACTCACTATAGGGGATGCCGAAGGAGAACGTATTA<br>TAATACGACTCACTATAGGGGTTTGCTGACCCTTGTCTTTATG	63<br>63	464
G protein-coupled receptor 161-like (GPCR)	XM_014438952.1	TAATACGACTCACTATAGGGCTCTGCTACTCCGCCATTTAC<br>TAATACGACTCACTATAGGGGGGTTCTGGCTTGAATCTCTT	65<br>62	472
Heat shock 70 kDa protein cognate 3 (HSP70)	XM_014425902.1	TAATACGACTCACTATAGGGGAGAGGGTTGAAGCTAGGAATG<br>TAATACGACTCACTATAGGGAGTAAGAGGAAACCGTGGAATG	63<br>63	487
Serine/threonine-protein phosphatase PP1-beta catalytic subunit (PP1)	XM_014431150.1	TAATACGACTCACTATAGGGGCGGCGATATACATGGACAATA<br>TAATACGACTCACTATAGGGCGGACCACAACAAATCACATAAC	63<br>64	438
Putative ATPase N2B (ATPase)	XM_014420983.1	TAATACGACTCACTATAGGGTGGGTGGATTGCCTGTTATT<br>TAATACGACTCACTATAGGGTTCCCTTGGGTGAGTTGATG	61<br>64	454
26S protease regulatory subunit 6B (26S)	XM_014421516.1	TAATACGACTCACTATAGGGCCTAGAGGCGTGCTAATGTATG<br>TAATACGACTCACTATAGGGCATCTTGGCCGTGATAGTAGAG	64<br>64	462
Dynamin	XM_014433358.1	TAATACGACTCACTATAGGGCAGGGATACACTTCCAGGATTAC<br>TAATACGACTCACTATAGGGGACCAGGTCGACACATTTAAGA	64<br>63	430
Uncharacterized Kanesin (Kanesin)	XM_014426093.1	TAATACGACTCACTATAGGGCTCCACCTCCTCCTCAGATAAA<br>TAATACGACTCACTATAGGGGCTGTCTTACTTTCAGGGTCTC	65<br>63	462

Table S2. List of Reference genes evaluated in this study

Symbol	Gene name	Accession number	Function
18S rRNA	18S ribosomal RNA	XM_014421522.1	Constituent of the small ribosomal subunit
EF1-α	Elongation factor 1 alpha	XM_014438029.1	Protein synthesis
Act	Actin	XM_014431329.1	Cytoskeletal protein
Ubi	Ubiquitin	XM_014429239.1	Protein degradation
60S RP	60S Ribosomal protein	XM_014430141.1	Cellular process of translation
β-Tubulin	Beta Tubulin	XM_014438117.1	Major constituent of microtubules

Table S3. List of primers used for qPCR study

Primer name	Primer sequence (5'-3')	Tm (^{0}C)	Product size (bp)
HhActin	F: GAGTGCATGAGACGACGTATT	55	112
	R: GCGATGCCTGGATACATAGTAG	55	
HhUbiquitin	F: GGATTACTGCCATGCTCAATTAC	54	110
	R: GACTATCGCACCAGGGTTAAA	55	
HhBeta Tubulin	F: TTACACAGAAGGTGCAGAGTTAG	54	103
	R: CAGGGAATGGGTCATCTGAAA	55	
Hh18S rRNA	F: TCGGAGGTCTTGTTGTTGATTAT	54	100
	R: CCTAAAGCCTGAGGTAATGGTG	55	
HhEF1 alpha	F: CCGCTAACATCACCACTGAA	55	111
	R: CCTTGACGGAGACGTTCTTTAC	55	
Hh60S RP	F: CCATCAGCAGCTTCTCTTATCA	54	119
	R: CTGGCGATGGTGAGGATTT	55	
HhATPase	F: ACCGATATTGCTGATGCTATGA	62	101
	R: AGATCATCAGGTGGACGATTG	62	
HhPP1	F: GCCCATCAGGTTGTAGAAGAT	62	93
	R: GTCAAACTCGCCACAGTAATTG	62	
HhGPCR	F: TCAGCGACATCTACATCAATCC	62	113
	R: GACGATGAACTACCACCAATCT	62	
HhIAP	F: GAAGTTGGTGATGACCCAGATA	62	106
	R: TGCCAGACTGATGTGCTATATT	62	
HhSNF7	F: CTTTGATGGCCGAAACTCTTG	62	100
	R: TCCGCACTTTCGATTTGAATTT	62	

Table S4. Descriptive statistics of candidate genes using BestKeeper software for dsRNA injected adult samples. **Abbreviations: GM**: geometric mean; **AM**: athematic mean; **Min** and **Max**: threshold values; **SD**: standard deviation; **CV**: coefficient of variation; **Max** [x-fold] and **Min** [x-fold]: threshold expression levels expressed as absolute x-fold over or under-regulated coefficient; **SD** [± x-fold] standard deviation of absolute regulation coefficient.

	Candidate gene					
**Factor**	***18SrRNA***	***Ubiquitin***	***Actin***	***60S RP***	***β-Tubulin***	***EF1-α***
**GM [CP]**	22.69	22.02	25.71	18.51	23.96	29.81
**AM [CP]**	22.70	22.06	25.72	18.53	25.40	31.87
**Min [CP]**	21.60	20.23	24.19	17.13	14.96	8.20
**Max [CP]**	23.94	27.15	27.28	22.56	39.90	39.90
**SD [+/- CP]**	**0.52**	**0.99**	**0.61**	**0.64**	**8.01**	**6.15**
**CV [% CP]**	2.27	4.50	2.37	3.47	31.54	19.30
**Min [x-fold]**	-2.12	-3.46	-2.86	-2.60	-510.91	-3198712.15
**Max [x-fold]**	2.38	35.00	2.97	16.59	63000.77	1090.63
**SD [+/- x-fold]**	1.43	1.99	1.52	1.56	258.20	71.03

Table S5. Descriptive statistics of candidate genes using BestKeeper software for dsRNA fed nymph samples. **Abbreviations: GM**: geometric mean; **AM**: athematic mean; **Min** and **Max**: threshold values; **SD**: standard deviation; **CV**: coefficient of variation; **Max** [x-fold] and **Min** [x-fold]: threshold expression levels expressed as absolute x-fold over or under-regulated coefficient; **SD** [± x-fold] standard deviation of absolute regulation coefficient.

	**Candidate gene**					
**Factor**	***18SrRNA***	***Ubiquitin***	***Actin***	***60S RP***	***β-Tubulin***	***EF1-α***
**GM [CP]**	20.92	17.76	24.12	15.76	30.29	29.63
**AM [CP]**	20.93	17.76	24.33	15.78	30.34	29.66
**Min [CP]**	19.77	16.92	18.39	13.47	27.06	27.35
**Max [CP]**	22.32	19.33	28.14	16.72	34.44	32.98
**SD [+/- CP]**	**0.51**	**0.43**	**2.52**	**0.52**	**1.36**	**1.14**
**CV [% CP]**	2.42	2.41	10.36	3.3	4.49	3.84
**Min [x-fold]**	-2.22	-1.78	-53.11	-4.9	-9.38	-4.86
**Max [x-fold]**	2.64	2.98	16.21	1.94	17.75	10.2
**SD [+/- x-fold]**	1.42	1.34	5.74	1.43	2.57	2.2

The sequence information and their accession numbers of *Halyomorpha halys* candidate genes used for dsRNA synthesis:

> HhIAP (Baculoviral IAP repeat-containing protein 7-B-like; Accession no: XM_014435389.1)
5'-
TATGCCCATTGGCCAGAAAATGCCCCTGTTGATGCTAGGAGAATTGCTGCTGCTG
GCTTTTATTACATGGGTCAAGGATTGGAAGTGCAATGTTTCAGTTGTGGTGGAAG
AATATCGGAATGGAATATAGGTGACAGTGTCATGTCTAAACACAGAGTACTAGA
CCCATTTTGTCCCTTTGTGCAAAACCCTATTTTGTCTGGTAATATACCACTTATTA
GTGATCGTAGAAATTCTCCTAATACTAGCCAGGAAAACCAATCAGTTTATCAAAA
TGAAAAATCAAGACTTGATACCTTTACAGCTTGGCCTATTCGCAGTATTGTTAGT
CCGGAGCTTCTAG-3'

> HhSNF7 (Charged multivesicular body protein 4b; Accession no: XM_014427464.1)
5'-
GGTCTGAGGAACAAGAGGTTAGCCCTTCAAGCTCTCAGGAGGAAGAAGAAGTTC
GAACAGCAATTGAAGCAGGTGGATGGGACGCTAGCGACTATAGAGCTGCAGAA
GGATGCTCTCGAAGGAGCGAGGACCAACACGGAGGTATGTGAGACCCTCAAGTT
GGCATCGGACGCCATGAAAGCGACGCAGAAGAACATAGACATAGACAAGTTGC
ACGACATATTGGACGACCTCAGCGAACAGCAGGAAATCGCCCAAGAGATTGCCG
ATGTCGTCTCTAGACCGATCAACATAGGTGGGGATGTTGACGACGATGAGTTAG
AACGTGAGCTGGAAGAGCTGGAGCAGGAAGAACTGGACAAAGAGATGATGGCC
ATTGGCGTCACGTCGGAGGATCTACCGGCAGTCCCTTCGGCTGCAGTGGCCGGAC
CAGCGACTTCTTCAACCCAAGAAGAGGACGACGAACTGAAG-3'

> HhActin (Actin-5C; Accession no: XM_014433214.1)
5'-
GGTTAAGGGACCGACAAAGATTTTCGTTGCCTCATAAACTATTAAGTATTTTTTA

TAATTGCATAGCATCTGCGAAGAAACATCTGGTTTTAAACAAAGAATAGTTTGGC
ATTCTTTTGGTAGCTACTACTGATACAGGATTTAAGATAATTTAAAAATATTTTGA
CATGTTACGTTTTTAATCGATTTATATTATAATCCACTTAATCAAAATCTAAATTT
AGCATTTACCTATTAATGGTTGATGATTTTTCACTTGTTACTATTAAAGGAATTTT
TTCTATCAAATGCTGCTTGTACATGAATACAAAATTTATGGTTGAGTGTTTACAA
AATTGTTAGTTTATAATTACCAGTAGTTATATAAAGATAATTTTAATTGTTGTCGC
CAAAATTTCTCTTGATCACTGAAACCTCTAATCTGATGATATATTATTCTAGCGGT
GAGTTGGGC-3'

> HhSRP (Signal recognition particle 54 kDa protein; Accession no: XM_014419857.1)
5'-
GATGCGTTGCGTTCGTTAAGTAATGCTACAATTATAAATGAAGAAGTTTTGGCTT
CTATGCTGAAAGAAGTATGTGCTGCATTATTGGAAGCTGATGTAAATATTCGTCT
TGTGAAAAAGTTACGTGAAAATGTCAGAGCTGTTATTGATTTCGATGAGATGGCT
GGTGGACTTAACAAGCGGCGAATGATACAAAGTGCTGTTTTTAAAGAATTAGTG
AAACTTGTTGATCCTGGTGTGAAGGCATTTCAACCTACTAAAGGAAAGCCAAAT
GTTATCATGTTTGTTGGTCTTCAAGGTTCAGGAAAAACAACTACTTGTACTAAAT
TAGCTTATCACTATTTAAAAAAAAATTGGAAAGCCTGTCTGGTATGTGCTGATAC
ATTCCGTGCTGGTGCCTACGATCAGTTAAAGCAGAATGCTACCAAGGCGAGGAT
TCCATTTTATGGAAGTTACACAGAAATTGACCCGGTTGTTATTGCTCAAGATGGT
GTGGA-3'

> HhNSF (Alpha-soluble NSF attachment protein; Accession no: XM_014434606.1)
5'-
ACACTGACATGGGAAGGTTTACAATGGCTGCAAAACATCATCAAACTATTGCTG
AAATGTATGAAACTGAAGCAGTTGATCTTGAAAGAGCTGTTCAACATTATGAGC
AAGCGGCTGATTTTTTTAGAGGAGAAGAAAGTAATTCTGCTGCAAATAAGTGTAT
GCTAAAAGTTGCTCAGTATGCTGCACAGTTAGAAAACTATGACAAAGCTATTCA
AATTTATGAGCAGGTCGCCTCTTCTTCCTTAGACAGTTCACTACTCAAGTACAGT
GCTAAAGAATATTTCTTCAGAGCTGCTTTATGTCATTTGTGTGTTGATGTACTTAA
TGCCCAGCAAGCTATGGAGCGATACATAGATCTTTACCCAACTTTCCAAGATTCC
AGAGAATATAAACTTTTGAAGGTTCTAATTGATCACATAGAGGAACAAAATGCT
GATGGCTTTACGGAAGCAGTCAAAGATTATGATTCTATTTCACGACTGGATCAGT
GG-3'

> HhROP (Protein ROP; Accession no: XM_014418690.1)
5'-
GATGCCGAAGGAGAACGTATTAAGGACCACATGAGAAATATTGTTCCTATACTC
CTCGACCAATCTGTCTCTAATTATGACAAGATGAGAATCATCCTCCTGTATACCC
TGTCCAAGAATGGTATTTCCGAAGAGAACTTGAACAAACTTGTCCAACACGCTCA
GATCCAGCCACATGAGAAACAGGCCATTGTCAATCTAGGAAATCTTGGCCTAAA
TGTTGTTGTTGATGGTACTCGTATCAAAAAGCCATATGTGCCTCCTCGTAAGGAG
CGTATTACTGAGCAAACATACCAGATGTCTCGTTGGACACCTGTGATCAAGGATC
TCATGGAGGATTGCATAGATGACAAGCTCGACCTGAAACATTTCCCCTTCCTGGC
TGGTAGGGCTGCGTCCTCAGGATATCATGCTCCCGCCAGCGTGCGATATGGACAC
TGGCATAAAGACAAGGGTCAGCAAAC-3'

> HhGPCR (G protein-coupled receptor 161-like; Accession no: XM_014438952.1)
5'-
CTCTGCTACTCCGCCATTTACACCGCCCTCACTCTACTCCTTCCGATAGCTGTCAT

CATGGCCTGTAACCTCAAGGTGCTGATGATTGCCCGATACCATAGACACAGGATC
GCTCGGGCCATCTTCGAGGTAACCCTATCAGCCCAAGTGACTATCACCCACCAGA
AGAATCCATTTGTTGTCTCTAGCGGATCAGGTCGTGGGAGGAGCCCTACAGCTAC
GGTACTTCAGCTGGTTGGCTCAGTCATCATACTGTACTCGCCCTACTACTGCATC
ATTCTGTGGCAGGGCTTAGGCAGGAAGCCTTCACCAGGGTTACTTGCAATTGCGT
CTAGTCTACTTGCTTGTGCACCAACACTCAACGGCCTTTTGTATGGCCTAAAGAA
CAAACTCCTTAGGCGAGCATTCAGACATTATTGGAGAAAGAAAATGACAAAGAA
TGAACTTGAACAAGAGATTCAAGCCAGAACCC-3'

> HhHSP70 (Heat shock 70 kDa protein cognate 3; Accession no: XM_014425902.1)
5'-
GAGAGGGTTGAAGCTAGGAATGAACTTGAATCGTATGCTTATTCTCTAAAGAATC
AGCTTTCTGACAAAGACAAACTTGGAGCAAAGGTCTCCGATGATGACAAGACTA
AGATGGAAGCAGCTATTGATGAAAAGATCAAGTGGTTAGAAGAAAACCAAGATG
CTGATAGTGAATCCTTCCAGCAACAGAAAAAAGAGCTTGAAGAAGTAGTTAAGC
CCATCATCACGAAACTTTACCAAGGTGGAGCACCTCCCCCAGGAGGAGCTGAAG
AAGATGTTAAGGATGAACTATAAGACTGCTTTAGCTTCATAATTCATTTTGTTTA
ACAAGACTTAAATTATTGAGAAAACAGCAGTATTTATATTTCTATATTTTGAAGT
AATTACTGCAATGTTGTTACTCTGTATAAAACCCAGTTAGAGGAAATTCTTATTT
ATTGAAATAATAATTAAAAAAAAAAAATCCATTCCACGGTTTCCTCTTACT-3'

> HhPP1 (Serine/threonine-protein phosphatase PP1-beta catalytic subunit; Accession no: XM_014431150.1)
5-
GCGGCGATATACATGGACAATATACAGATCTCCTTAGGTTATTTGAATACGGTGG
TTTTCCGCCTGAAGCAAATTATCTGTTTTTGGGTGATTATGTCGATCGTGGAAAAC
AGTCTCTTGAAACCATTTGCCTTCTACTTGCATATAAAATCAAATATCCAGAAAA
TTTCTTTCTTCTTCGAGGAAACCACGAATGTGCATCAATCAACAGAATATATGGA
TTTTATGATGAATGTAAAAGGAGGTATAACATAAAGTTATGGAAAACATTCACTG
ATTGTTTCAACTGTCTTCCAATCGCTGCCATTATTGATGAAAAAATATTTTGTTGC
CATGGAGGTTTAAGTCCAGATTTGCAAGGAATGGAACAAATTAGACGAATAATG
CGACCTACTGATGTTCCGGACACGGGTCTGTTATGTGATTTGTTGTGGTCCG-3'

> HhATPase (Putative ATPase N2B; Accession no: XM_014420983.1)
5'-
TGGGTGGATTGCCTGTTATTCTTGTGAGATGGTTCTTAAATTATATAAAACTTCTT
TTATTTCAGTTTTTAATATGTGATATTTTCCTTGTTTAATGACAAAATAACAATTG
CCCTAAGATCTATTATGTTACCATTAACTTATTTAATATTTTCTACAAATCAAAAG
TTCCTTAAAGTTTCTACTAGTCCCAGCTTTTATCATTTGTTTAAAGAAAAATTTTC
AGTTCAAGTATTGAAGCATCTAAACAGTGCTGTTCAACCCTCACTGAGTCCAATC
CAAGCTCTTGAAACATTGATAACAAAAGGTGAATTAAGAGAAGATCCCCATCAA
AAAATTATTGTTCAAGAACTTGAGAAGGTTTATTTAGATATCCAGAACTATTCCC
CAGCTAAAGTAGGATTTTTTGCTAAATTATTCCATCAAAGCAATGCCATCAACTC
ACCCAAGGGAA-3'

> Hh26S (26S protease regulatory subunit 6B; Accession no: XM_014421516.1)
5-
CCTAGAGGCGTGCTAATGTATGGGCCACCAGGCTGCGGCAAAACAATGCTTGCT
AAAGCCGTTGCCCATCATACCACTGCGGCGTTCATCCGAGTAGTTGGATCTGAAT
TCGTTCAAAAATATCTTGGAGAAGGACCTAGAATGGTTCGTGACGTTTTCAGACT

CGCTAAAGAAAACGCTCCTGCCATCATCTTCATCGACGAAATTGATGCCATTGCT
ACAAAAAGGTTTGATGCTCAGACTGGTGCTGACAGAGAAGTGCAGAGGATTCTA
TTGGAACTGCTCAACCAGATGGATGGTTTTGACCAAACCACGAATGTAAAGGTT
ATAATGGCTACAAACAGAGCAGATACCTTGGACCCTGCCCTGCTAAGGCCCGGT
AGGCTGGACAGGAAGATAGAATTCCCTCTTCCTGACAGGCGGCAGAAGAGGCTC
GTCTTCTCTACTATCACGGCCAAGATG-3'

> HhDynamin (Dynamin; Accession no: XM_014433358.1)
5'-
CAGGGATACACTTCCAGGATTACGAGATAAACTCCAGAAACAAATGCTTGCCTT
AGAAAAAGATGTTGAACAGTACAAATACTTCAGACCTGATGACCCTTCTATCAA
AACCAAAGCAATGCTGCAGATGATACAACAGTTACAGTCGGATTTTGAAAGAAC
CATTGAAGGGAGTGGTTCAGCCCAAATCAACACTGTGGAGCTGTCTGGTGGTGCT
AAGATTAACCGGCTCTTTCATGAGAGGTTTCCTTTTGAACTGGTCAAAATGGAAT
TCGATGAAAAGGAACTCCGAAGGGAAATTGCTTTTGCTATCAGAAATATTCATGG
TATAAGAGTTGGCTTATTTACACCTGATATGGCCTTTGAAGCAATAGTCAAAAAG
CAGATTGAAAGGCTCAAAGAACCATGTCTTAAATGTGTCGACCTGGTC-3'

> HhKanesin (Uncharacterized Kanesin; Accession no: XM_014426093.1)
5'-
CTCCACCTCCTCCTCAGATAAATGGATTTGAACCCTCAGTGGCACCTCCTGGTTTC
ATTAAACAAAATCTTCCTGGTGAAATTCAACAAGGATTAATCAGACCTGTACTTC
CTGTCACTTACTTTAACCCAGATTACCAATTATACAATGGCACACCAGGTGTTGA
ACCATCAGTTGATGTTTTAACAAGTCTCATGACACCACCAACAGCTCCTAATTTC
TCTCAGGTGCCTACTCAAAATTATGTTGTTGAACCATCTCAAGAAAACACTGCTG
CATCTGCTACACCAGCAGCTATCACAGCTTCAACTCCTGATATAAATGTTAGTGA
ATTGTATAAAAAACTAGTAGCTATTGGAGTAGTGCCTCCATTAAAAGAAAAAGA
AAAGGAGAATACTGAAAATAGGTTAGCTGTTAAACCAGTTGATTTTTCGAAGCCT
GAGACCCTGAAAGTAAGACAGC-3'
